# Formulation of pH-Responsive Methacrylate-Based Polyelectrolyte-Stabilized Nanoparticles for Applications in Drug Delivery

**DOI:** 10.1021/acsanm.2c04539

**Published:** 2022-11-24

**Authors:** Bumjun Kim, Dawei Zhang, Madeleine S. Armstrong, István Pelczer, Robert K. Prud’homme

**Affiliations:** † Department of Chemical and Biological Engineering, Princeton University, Princeton, New Jersey08544, United States; ‡ Department of Chemistry, Princeton University, Princeton, New Jersey08544, United States

**Keywords:** flash nanoprecipitation, nanoparticles, enteric-coating polymers, pH-responsive polymers, polyelectrolytes, methacrylate-based copolymers, drug delivery, Eudragit

## Abstract

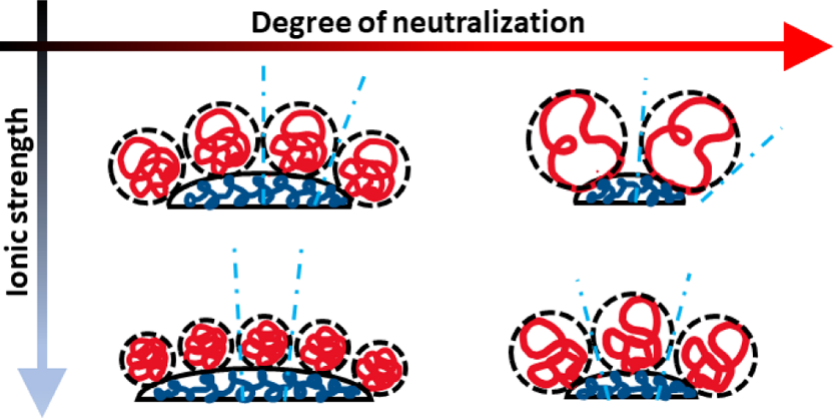

pH-responsive polyelectrolytes, including methacrylate-based anionic copolymers (MACs), are widely used as enteric coatings and matrices in oral drug delivery. Despite their widespread use in these macroscopic applications, the molecular understanding of their use as stabilizers for nanoparticles (NPs) is lacking. Here, we investigate how MACs can be used to create NPs for therapeutic drug delivery and the role of MAC molecular properties on the assembly of NPs via flash nanoprecipitation. The NP size is tuned from 59 to 454 nm by changing the degree of neutralization, ionic strength, total mass concentration, and the core-to-MAC ratio. The NP size is determined by the volume of hydrophilic domains on the surface relative to the volume of hydrophobic domains in the core. We calculate the dimensions of the hydrophobic NP core relative to the thickness of the polyelectrolyte layer over a range of ionizations. Importantly, the results are shown to apply to both high-molecular-weight polymers as core materials and small-molecule drugs. The pH responsiveness of MAC-stabilized NPs is also demonstrated. Future development of polyelectrolyte copolymer-stabilized nanomedicines will benefit from the guiding principles established in this study.

## Introduction

The translation of research to clinical application for polymeric nanoparticles (NPs) is often confounded by the requirement that FDA-approved polymers be used in the formulation. A further limitation for orally delivered, non-parenteral drugs is the cost of the polymers involved. Block copolymers, such as polylactic acid-*b*-polyethylene glycol, which are FDA-approved, have costs that are often too high to warrant their use in oral formulations. In this paper, we present the use of FDA-approved and relatively low-cost, amphiphilic, methacrylate-based anionic copolymers (MACs) and modified cellulosic polymers to prepare NPs. The MAC polymers are sold under the trade name of Eudragits and are widely used in tablet formulations for enteric-coating of tablets or for making bulk pH-responsive matrices. MACs have also been utilized as a stabilizer or co-stabilizer in the formulation of NPs or microparticles (MPs).^1−8^ The size and surface chemistry of particles impact the distribution within the GI tract. In murine colitis models, conventional micronized particles (>1 μm) did not effectively penetrate the mucus mesh.^1,9,10^ In contrast, NPs (<500 nm) exhibited improved penetration of the mucus layer; therefore, the nanoscale dimensions of these NPs may increase the local drug concentration in inflamed epithelial tissues.^1,10,11^ Therefore, facile tuning of particle size as well as surface chemistry offer the possibility to deliver the drug in closer proximity to epithelial surfaces. Surprisingly, despite the extensive efforts in the development of oral and rectal delivery systems, mechanistic understanding of how these methacrylate-based and cellulose-based polyelectrolyte polymers can be formulated into NPs is limited.

We present NP formation using a kinetically controlled precipitation process, flash nanoprecipitation (FNP). FNP is a process whereby a water-miscible solvent stream containing hydrophobic core substituents and amphiphilic stabilizers is mixed under turbulent flow conditions against an aqueous anti-solvent stream. The confined impinging jet mixers produce mixing times faster than particle formation times (1.5–3 vs 10–100 ms).^12−14^ Homogeneous and rapid mixing at high supersaturation promotes uniform nucleation, while growth is limited by the formation of the stabilizing corona.^15,16^ As a continuous process, FNP enables the scalable production of NPs from the bench scale to the industrial scale.^17^

Amphiphilic stabilization requires proper balancing of the size of hydrophilic and hydrophobic domains.^18^ For polyelectrolyte polymers, this balance can be controlled by the degree of ionization and the ionic strength of the aqueous media.^19,20^ Herein, we systemically investigate the impact of formulation parameters on NPs stabilized by MACs. We demonstrate the capability to control particle size from 59 to 454 nm using a model hydrophobic core and MACs. We also demonstrate that commonly used random copolymers with different chemistry, such as cellulose-based anionic copolymers (CACs), hydroxypropylmethylcellulose acetate succinate (HPMCAS-HF), hydroxypropylmethylcellulose phthalate (HP-55 and HP-55s), and methacrylate-based cationic copolymers (Eudragit RL-PO), can be similarly employed. Our results will serve as guidelines for the scalable production of functional NPs stabilized by ionic copolymers, which can be applied to formulations of oral or rectal delivery systems.

## Experimental Section

### Materials

Poly(lactic acid) (PLA) (RESOMER R 203 S) was purchased from Evonik Industries (Essen, Germany). Lumefantrine (LMN) was obtained as a gift from Medicines for Malaria Ventures. Eudragit S100, Eudragit L100, Eudragit L100-55, and Eudragit RL-PO were gifts from Evonik Industries. HP-55 and HP-55s were gifts from Shin-Etsu Chemical (Tokyo, Japan). HPMCAS-HF (AquaSolve) was a gift from Ashland Global Holdings (Delaware, US). Sodium hydroxide, hydrochloric acid, sodium chloride, acetonitrile (ACN), dimethyl sulfoxide (DMSO), trifluoroacetic acid (TFA), and tetrahydrofuran (THF) were obtained from Fisher Scientific (Massachusetts, US). BODIPY 650/665-X NHS Ester (BDP 650/665) was a gift from ThermoFisher Scientific (Massachusetts, US). MidiKros hollow fiber filter modules were acquired from Repligen (Massachusetts, US). Nanosep Omega Membrane 300 K filters were purchased from Pall Corporation (New York, US). Deuterium oxide (*d*, 99.96%) was acquired from Cambridge Isotype Laboratories (Massachusetts, US). Sodium 3-(trimethylsilyl)-1-propanesulfonate (DSS) was purchased from TCI America (Oregon, US). Calcium chloride dihydrate, iron(III) chloride hexahydrate, and methacrylic acid were obtained from MilliporeSigma (Massachusetts, US). Salts and buffers for the fed-state intestinal fluid (FeSSIF) were purchased from Biorelevant (London, England).

### Synthesis and Characterization of NP

**Scheme 1:**
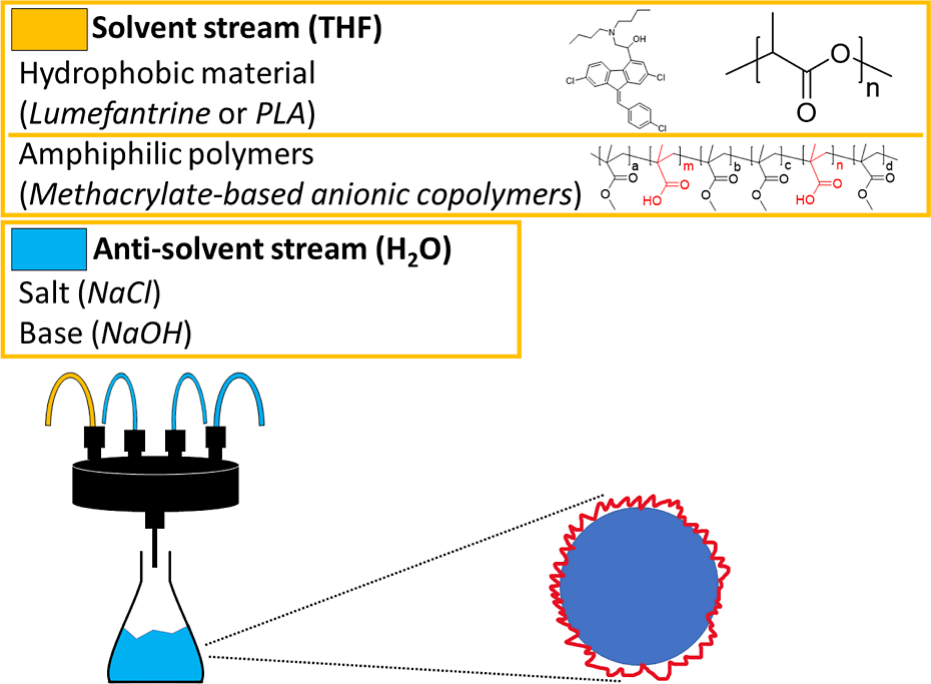
Graphical Illustration of the FNP Process

Rapid precipitation and NP formation were achieved with the micro multi-inlet vortex mixer (μMIVM) as has been described previously.^14,21^ Formulation parameters are shown in Scheme 1. Briefly, a solvent stream contains core materials and polyelectrolytes dissolved in THF, and the anti-solvent streams are water. PLA (log *P* = 10)^22^ or LMN (log *P* = 9.2)^23^ were selected as core materials due to their hydrophobicity, giving an aqueous solubility of less than 80 ng/mL.^24^ Different polyelectrolytes were used as stabilizers as shown in Tables 1 and S1. Unless indicated, the four inlet streams of the μMIVM are set at equal flow rates (60 mL/min) with one stream introducing the core material and the polyelectrolyte polymer in THF and the other three streams introducing aqueous anti-solvents. This produces an exit stream with 75% anti-solvent (H_2_O). A mixer Reynolds number of approximately 46,000 was used in this study. To increase the supersaturation, in certain cases, the flow rate of anti-solvent streams was increased to make 91% H_2_O in the final mixed solvent stream. NPs eluted through the exit stream at 75% anti-solvent are further diluted with additional H_2_O in receiving vials, so the final THF/H_2_O ratio would be 10:90. To alter the degree of neutralization (DN) and ionic strength, designated amounts of NaOH and NaCl were added in the anti-solvent H_2_O streams. The total mass concentration (TMC) is noted as the total solids (mg/mL) dissolved in the THF stream. This was varied by changing the concentration of solute mass in the solvent stream. NPs synthesized via FNP were further diluted 10-fold in 15 mM NaCl solution before dynamic light scattering (DLS) measurements. For surface charge measurement, samples were diluted in different concentrations of NaCl solution to obtain a conductivity of 2 mS/cm. Dilution was performed to achieve dilute enough dispersions to avoid multiple scattering and to have all samples with a common ionic strength for the ζ potential measurements. Size and surface charge were measured with a Zetasizer Nano-ZS (Malvern Instruments, Malvern, U.K.). The detection angle for the measurement was 173° with a helium-neon laser (632 nm).

**Table 1:** Characteristics of Commercially Available Enteric Polymers

name	Mw (kDa)	ionizable group (wt %)	polymer	dissolution pH
Eudragit S100	125^a^	27.6–30.7^a^ (methacrylic)	methacrylic acid–methyl methacrylate^a^	7^26^
Eudragit L100	125^a^	46.0–50.6^a^ (methacrylic)	methacrylic acid–methyl methacrylate^a^	6^26^
Eudragit L100-55	250^b^	46.0–50.6^b^ (methacrylic)	methacrylic acid–ethyl acrylate^b^	5.5^26^
HP-55	37.9^c^	21.0–27.0^d^ (phthalyl)	hydroxypropylmethylcellulose phthalate^d^	5^d^
HP-55s	45.6^c^	27.0–35.0^d^ (phthalyl)	hydroxypropylmethylcellulose phthalate^d^	5.5^d^
HPMCAS-HF	10–500^e^	4–8^e^ (succinyl)	hydroxypropylmethylcellulose acetate succinate^e^	6.8^e^
Eudragit RL-PO	32^f^	8.9–12.0^f^ (ammonio) permanently charged	ammonio methacrylate copolymer^f^	non-pH responsive^f^

a Technical information sheet (INFO 7.3/E) from Evonik Industrial AG.

b Technical information sheet (INFO 7.4/E) from Evonik Industrial AG.

c Technical information (H-008, molecular weight of HPMCP) from Shin-Etsu Chemical Co., Ltd.

d Hypromellose phthalate NF brochure (2014.5/700) from Shin-Etsu Chemical Co., Ltd.

e AquaSolve HPMCAS-HF physical and chemical properties’ handbook from Ashland.

f Technical information sheet (INFO 7.7/E) from Evonik Industrial AG.

### Demonstrating the Scalability of FNP

NPs are synthesized at 5 or 500 mL scales through either FNP or conventional nanoprecipitation. The base formulation has one organic stream consisting of 5 mg/mL of PLA and 5 mg/mL of S100 dissolved in THF and three aqueous streams consisting of 125 mM of NaCl and 1 equiv amount of NaOH relative to the carboxyl group in S100. All the experiments in this section employed the same formulation but different mixing processes (e.g., FNP vs the conventional nanoprecipitation method).

To make a 5 mL scale of NP suspensions via FNP, a μMIVM was used as described above. To make a 500 mL scale, pump-driven MIVM was used. One organic stream and three aqueous streams are set at equal flow rates (50 mL/min), which corresponds to the mixer Reynolds number of 15,800. NPs eluted through the exit stream at 75% anti-solvent are further diluted, so the final THF/H_2_O ratio would be 10:90.

To make 5 or 500 mL scale of NP suspension via conventional FNP, the organic stream was injected into aqueous solution via a 1 mL syringe or a 60 mL syringe. The aqueous solution was stirred with a magnetic stir bar at 200 rpm on the stir plate during solvent injection. The final THF/H_2_O ratio is 10:90.

### Investigating the DN by ^13^C NMR

Quantitative ^13^C NMR spectra were recorded on a Bruker Avance-III 500 MHz spectrometer, equipped with a C–H dual cryoprobe optimized for ^13^C-detection and a BACS-120 autosampler in solutions of THF/H_2_O with/without varying degrees of neutralization at methacrylic acid 2% v/v. A pair of coaxial NMR tubes were used. Specifically, a 300 μL sample was loaded into the 4.1 mm OD inner tube; the 5 mm OD outer tube contained 50 μL D_2_O solution and 5 mg/mL of DSS for deuterium-locking, shimming, and chemical shift reference, respectively. Typical acquisition parameters were a 28 s relaxation delay and 120 repeated scans at a 295 K controlled temperature. Data were processed using MestReNova (v.14.2, Mestrelab Research S.L., Santiago de Compostela, Spain).

### Titration of Pre-formed NPs

To simulate the pH responsiveness of NPs through the gastrointestinal tract, the size and ζ potentials were monitored during acid and base titrations. NPs were synthesized with excess DN (150%, i.e., highly negatively charged), ionic strength at 125 mM, the core/stabilizer ratio at 1:1, and TMC of 10 mg/mL. Initially, NPs were titrated with HCl until the suspension contained 50% excess HCl (−50% DN), relative to the number of carboxyl groups in S100. Then, the NP suspensions were titrated with NaOH until all the carboxyl groups in S100 were fully deprotonated (100% DN). At each DN level, pH was measured with a pH-meter (Accumet Research AR-20, Fisher Scientific, Waltham, MA), and 0.1% v/v of the sample was taken to measure the size and ζ potential using Zetasizer Nano-ZS.

### Lyophilization of NPs

Freshly prepared LMN-encapsulated NPs obtained via FNP were diluted with threefold deionized water. A hydrophilic polymer, HPMC E3, was added at 1 equivalent mass of NPs as a cryoprotectant; the final THF concentration was approximately 3% v/v. This protocol was based on our previously optimized lyophilization for LMN using HPMCAS stabilizers.^25^ The mixture was frozen in liquid nitrogen and lyophilized at −20 °C under vacuum using a VirTis Advantage lyophilizer (Gardiner, NY).

For the lyophilization of BDP 650/665-encapsulated NPs, THF was first removed by dialysis. NP suspensions were dialyzed against 100-fold excess water (2.7 mM NaOH) twice for 2 h. Dialyzed NP suspensions were concentrated 10-fold via a MIDIKROS tangential flow filtration unit (Repligen, D02-E100-05-S). Concentrated NPs were combined with 5 wt % trehalose and freeze-dried, as described above. Lyophilized powders were resuspended in double-distilled water. NP size was measured via DLS as described above.

### Measuring the Encapsulation Efficiency and Release of LMN

To measure the total LMN, freshly prepared NP suspensions were dissolved in 50-fold volume of DMSO to completely dissolve the NP and release LMN. LMN was measured through a high-performance liquid chromatography (HPLC) system, Agilent 1100 equipped with a Gemni 5 μm C18 column (150 × 4.6 mm). To measure the unencapsulated LMN, Nanosep omega filters (300 kDa) were used to collect the filtrate through centrifugation at 3000*g* for 10 min. The LMN concentration in filtrates was measured via HPLC, as previously described.^16^ An isocratic mobile phase, composed of ACN/water at 60:40 with 0.05% TFA, was applied to elute LMN at a flow rate of 1 mL/min. A LMN peak was detected at 347 nm at 6.8 min. The encapsulation efficiency (EE %) is defined as
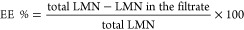
1

To measure the release kinetics of LMN, lyophilized NP samples were first resuspended in deionized water at 500 μg/mL of LMN. NP suspensions were then diluted 10-fold in fasted state simulated gastric fluid (FaSSGF) and incubated in an incubator shaker (Innova 4080, New Brunswick Scientific) for 0.5 h at 37 °C. NPs in FaSSGF were further diluted 10-fold in fasted state simulated intestinal fluid (FaSSIF) and incubated in the orbital incubator shaker for an additional 6 h. Aliquots were sampled at 0.25, 0.5, 1, 2, 3, and 6 h post-incubation. To separate soluble LMN from LMN-encapsulated NPs, samples were centrifuged at 21,000*g* for 10 min. Supernatants were frozen and lyophilized. Lyophilized supernatants were dissolved in a mixture of THF/ACN at a 10:90 ratio, and insoluble salts were filtered through 0.1 μm PTFE Whatman syringe filters (GE Healthcare Life Sciences). LMN in the supernatant was measured via HPLC, as described above.

## Results and Discussion

### Effect of the DN, Ionic Strength, Percent Hydrophobic Core, and TMCs on the Formation of Polyelectrolyte Polymer-Stabilized NPs

A μMIVM^14^ was used to produce NPs coated with methacrylate-based copolymers or functionalized anionic cellulose polymers as described in Scheme 1. The polymers and chemical structures used in our study are summarized in Tables 1 and S1. Formulation variables and conditions are presented in each figure. The size distributions of NPs produced via FNP at 5 and 500 mL scales exhibit a minimal difference (avg NP size. 134 vs 148 nm) (Figure S1A). On the other hand, a clear peak shift in size distribution is observed when conventional nanoprecipitation is employed to produce NPs at two different scales (avg NP size. 219 vs 158 nm) (Figure S1B). The size difference would be even greater in batch precipitations if clinical scale volume (10–1000 L) was required. This demonstrates the scalability of FNP, which cannot be achieved via a conventional nanoprecipitation process.

**Figure 1:**
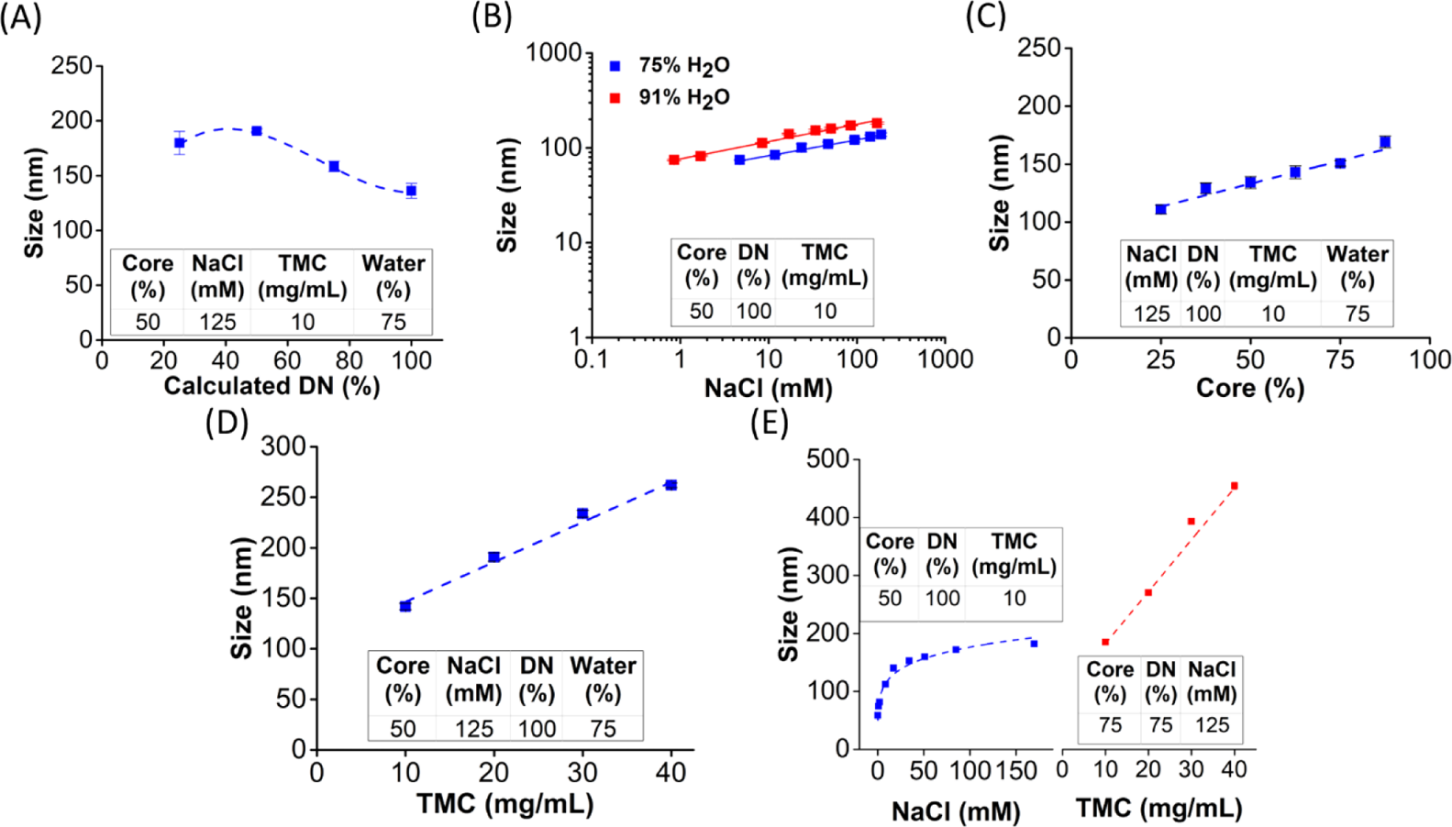
Controlling the formation of S100-stabilized NPs. The impact of DN of the polymer (A); ionic strength (B); percent core (C); and TMC (D) on NP size. The size of S100-stabilized NPs encapsulating PLA could be tuned over the range from 59 to 454 nm by changing the ionic strength, DN, percent core, and TMC (E). Sizes of NPs are determined by their diameters measured by DLS. Red data points in (B) were replotted with an additional data point at NaCl = 0 mM and presented as the first section of the graph in (E). This is expressed as mean ± SD from three measurements. The rest of the formulations were independently synthesized thrice, and the data are expressed as mean ± SEM (*n* = 3).

To stabilize NPs, three MACs were used: Eudragit S100 (S100), Eudragit L100 (L100), and Eudragit L100-55 (L100-55). S100 has 30 wt % ionizable methacrylic acids (MAAs), whereas L100 and L100-55 have 50 wt % MAAs. S100 and L100 have similar molecular weights (125 kDa), whereas L100-55 has a higher molecular weight (250 kDa). All three polymers readily dissolve in THF, the solvent for the core material. S100 produced smaller particles compared to L100 and L100-55 (Figure S2A); therefore, S100 was chosen for further studies. PLA, an FDA-approved, generally recognized as safe (GRAS) polymer, was utilized as the model hydrophobic core material. The impact of the DN, ionic strength, core-to-stabilizer ratio, and TMC on NP formations were examined. Physicochemical properties of NPs in Figure 1 are tabulated in Table S2.

#### Effect of DN

**Scheme 2:**
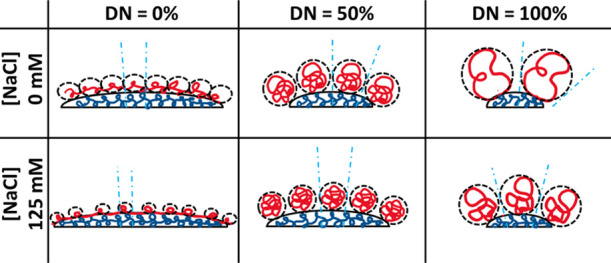
Increase in the NP Surface Curvature with Increased Volume of the Hydrophilic Domain as a Function of DN and Ionic Strength of Media

Using S100 and PLA, we investigate the effect of DN. Increasing the DN of S100 results in a decrease in NP size (Figure 1A). The DN reported is based on the stoichiometry of the number of acid groups on the MAC and the amount of NaOH added in the anti-solvent stream. The salt form of S100 cannot be dissolved in THF; the acidic form of S100 was molecularly dissolved in the THF stream. During micromixing, MAA is ionized, and the hydrophobic methyl methacrylate (MMA) groups bind to the hydrophobic core material. The NPs grow by aggregation of these core/copolymer clusters. The growth stops when the steric stabilization of the corona layer is dense enough to prevent further cluster aggregation. The ionized MAA groups provide stabilization by inter-particle electrostatic and steric repulsion. The sizes of the final NPs are determined by the volume of hydrophilic domains on the surface versus the volume of hydrophobic domains in the core, as shown schematically in Scheme 2. The concept is similar to the concept of the “packing parameter” used to describe surfactant self-assembly.^27^ The relative volume of the hydrophilic domain increases, and the volume of the hydrophobic domain decreases with increasing DN. When a PLA core is present, the NP size is 191 nm at 50% DN and it decreases to 142 nm at 100% DN. At DN = 0, the NPs failed to form due to insufficient charge or volume of ionized chains on their surfaces to prevent aggregation; bulk precipitates result.

In the absence of a core, during anti-solvent mixing, the S100-dissolved polymer collapses. As shown in Figures S2B,C, at 25% DN, the hydrophobic chain collapses into a unimolecular micelle with a size on the order of 25–100 nm but a high photon count rate. A high photon count rate indicates a dense core. Similar-sized NPs are formed at 50% DN but with lower optical scattering intensity due to the solvation of polymer chains in the core. At 75 and 100% DN, polymers are highly solvated and associated into large swollen gel MPs; the sizes are estimated to be as large as 30 μm based on correlograms. These data show how the change in the balance of hydrophilic and hydrophobic domains in S100 impacts the state of polymer assembly in the absence of core materials. The addition of PLA acts as a nucleation agent and templates the formation of NPs with a dense core with the associated polyelectrolyte chains on the NP surface.

#### Effect of Ionic Strength

NP size increases with increasing ionic strength in the anti-solvent stream during precipitation. The DN, core percentage, and TMC were fixed at 100%, 50 wt %, and 10 mg/mL, respectively. Two levels of THF/H_2_O ratios were used in the precipitations: 25:75 and 9:91. NPs are produced with diameters of 84 and 59 nm without the salt screening at 75 and 91% of H_2_O. The sizes of NPs precipitated with 170 mM of NaCl are 133 and 193 nm (Figure 1B). With increasing ionic strength, the NP size increases according to the power laws of 0.17 and 0.18 for 75% vol and 91% vol water, respectively. Salt screens ionic repulsions and decreases the volume of the hydrated chain domains.^28^ This leads to a lower surface area occupied by the hydrophilic domains and a correspondingly larger packing parameter (i.e., a larger NP radius of curvature). PDI tends to decrease as ionic strength increases (Table S2); NPs exhibit more homogeneous size distribution at higher ionic strengths.

#### Effect of the Core-to-Stabilizer Ratio

NP size increases as the ratio of the PLA core concentration to the stabilizing polymer concentration increases (Figure 1C). NP size increases from 111 nm at 25% core ratio to 169 nm at 87.5% core ratio. Increasing the core loading leads to a larger core volume relative to the number of stabilizing hydrophilic domains, so the radius of the NP increases. NPs are also more homogeneous at higher core loadings, as indicated by a lower PDI (Table S2).

#### Effect of Total Mass Concentration (TMC)

NP size increases as the TMC increases (Figure 1D). The TMC is the sum of the concentrations of the core material and the stabilizing copolymer in the organic solvent stream. NP size increases from 142 to 262 nm as mass concentration increases from 10 to 40 mg/mL in the precipitation step. This observation of increasing size with increasing concentration is explained by the increase in collision probability as previously observed with block copolymers.^18^ PDI also decreases as TMC increases (Table S2).

We have demonstrated that the sizes of S100-stabilized NPs can be controlled from 59 to 454 nm by tuning formulation variables, as shown in Figure 1E. The DN and salt concentration tune size by controlling the volume of the hydrophilic domain of the stabilizing polymer. Figure 1E shows NPs from 50 to 200 nm with varying ionic strengths for the single formulation: 100% DN, 50% core loading, and 10 mg/mL of TMC at a THF/H_2_O ratio of 9:91. If the DN is reduced to 75% and the core-to-stabilizer ratio is increased to 75%, the size can be tuned from 200 to 450 nm by increasing the TMC from 10 to 40 mg/mL. This demonstrates that understanding the physicochemical phenomena that control particle assembly enables the rational design of NP properties.

### Mechanistic Analysis on the Deprotonation of MAA

The results on the effect of DN and ionic strength on NP size demonstrate the role that electrostatics plays in the NP aggregation process, as shown in Figure 1A,B. When S100 chains are first exposed to water during FNP, the MAA monomers become ionized, and the MMA monomers begin precipitating. Ionization/deprotonation of the acid groups can depend on the polarity and hydrogen bonding properties of the media. Therefore, to determine the actual DN, we used NMR where the chemical shifts of ^1^H NMR or ^13^C NMR of ionizable groups can be used to directly measure the DN in mixed solvents.^29,30^ Preliminary experiments to measure the chemical shifts of MAA groups in S100 using ^13^C NMR proved infeasible. At higher water ratios, the S100 precipitated in the mixed solvents, and the relaxation times of MAA groups became too broad to enable quantification. Therefore, we use MAA monomers as a surrogate for the polymer chain. Although there are limitations with this as a model, the MAA monomer remains soluble over the entire range of solvent ratios and DNs.

**Figure 2:**
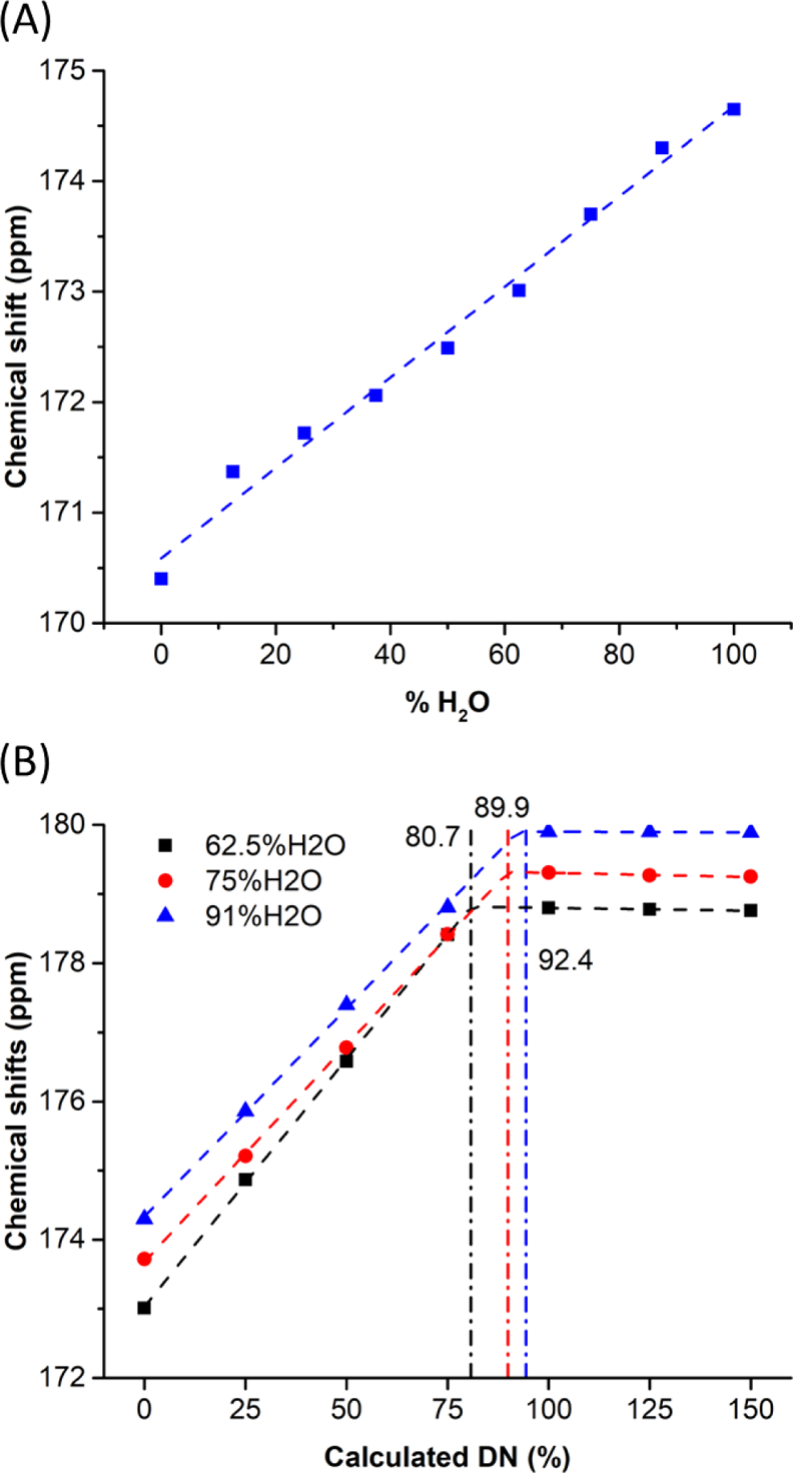
Chemical shift changes of the carboxyl ^13^C resonance in MAA. (A) Ratio of THF/H_2_O was varied from 100:0 to 0:100. (B) DN was varied from 0 to 150% at 37.5:62.5, 25:75, and 9:91 ratios of THF: H_2_O. Vertical lines indicate the points of saturation of chemical shifts at each condition.

The data in Figure 2A show the chemical shift of MAA changing from 170.4 ppm in pure THF to 174.7 ppm in water. THF is aprotic; therefore, deprotonation of MAA does not occur in pure THF. With the addition of water, the signal from the ^13^C in the COOH shifts downfield. This occurs due to the changes in hydrogen bonding patterns among THF, H_2_O, and MAA. In addition, spontaneous ionization occurs even without the addition of OH^–^, which is consistent with the p*K*_a_ of MAA. FNP occurs in the THF/H_2_O ratio of 1:1 to 1:9. Over this range, the chemical shifts are linear with the THF/H_2_O ratio.

The neutralization of the MAA group in different solvent mixtures (62.5, 75, and 91% H_2_O) is shown in Figure 2B. The shift is entirely linear between DN = 0 and DN = 75%, regardless of the ratio of THF/H_2_O in solvent mixtures. This linearity is consistent with quantitative neutralization by the added OH^–^. The chemical shift of MAA at 62.5, 75, and 91% H_2_O starts at 173.01, 173.72, and 174.30 ppm, respectively; saturation occurs at chemical shifts of 178.80, 179.31, and 179.90 ppm, respectively. The intersection points of the ionization curve and the saturation curve occur at 80.7, 89.9, and 92.4% of the calculated DN based on OH^–^ addition. The saturation of the chemical shift indicates complete deprotonation of MAAs. Therefore, S100 precipitated at both 75% H_2_O and 91% H_2_O will be completely neutralized when the amount of NaOH required for 100% calculated DN is added. Applying this result to MAA groups in S100 chains, MAAs would also be completely neutralized and not likely trapped inside the hydrophobic core of the NPs during FNP; solvent intermixing time (1.5 ms) is faster than the NP assembly time (20–50 ms).^12,18,31^ These NMR data show that differences in sizes of NPs produced at different THF/H_2_O ratios (Figure 1B), despite the same amount of OH^–^ addition, are not due to differences in actual DN but rather changes in electrostatic forces. The electrostatic force between charges is inversely proportional to the dielectric constant according to Coulomb’s Law.^32^
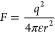
2where *F* is the repulsive force, *q* is the charge, *r* is the distance between the charges, and ε is the dielectric constant of the solution. Increasing the dielectric constant decreases the repulsive force. Equation 2 represents the force between individually charged monomer units, which will control the “blob” size of the steric stabilizing layer on the surface of the NP (Scheme 1). The interactions between NPs themselves would involve an inter-particle analysis of the London-van der Waals and electrostatic forces between particles. We have partially addressed this in a previous study on a simpler NP system,^28^ but a detailed analysis of NPs with polyelectrolyte brushes is beyond the scope of this paper and not needed to understand the phenomena involved.

As the THF/H_2_O ratio changes from 25:75 to 9:91, the dielectric constant increases from 60.5 to 71.8 (at RT),^33^ which results in a decrease in electrostatic repulsion between ionized MAA units of S100. Decreased repulsion means that core/S100 clusters aggregate more rapidly, resulting in larger NPs. Additional NMR data and further discussion can be found in Figure S3.

### Investigating pH Responsiveness of S100-Stabilized NPs

**Figure 3:**
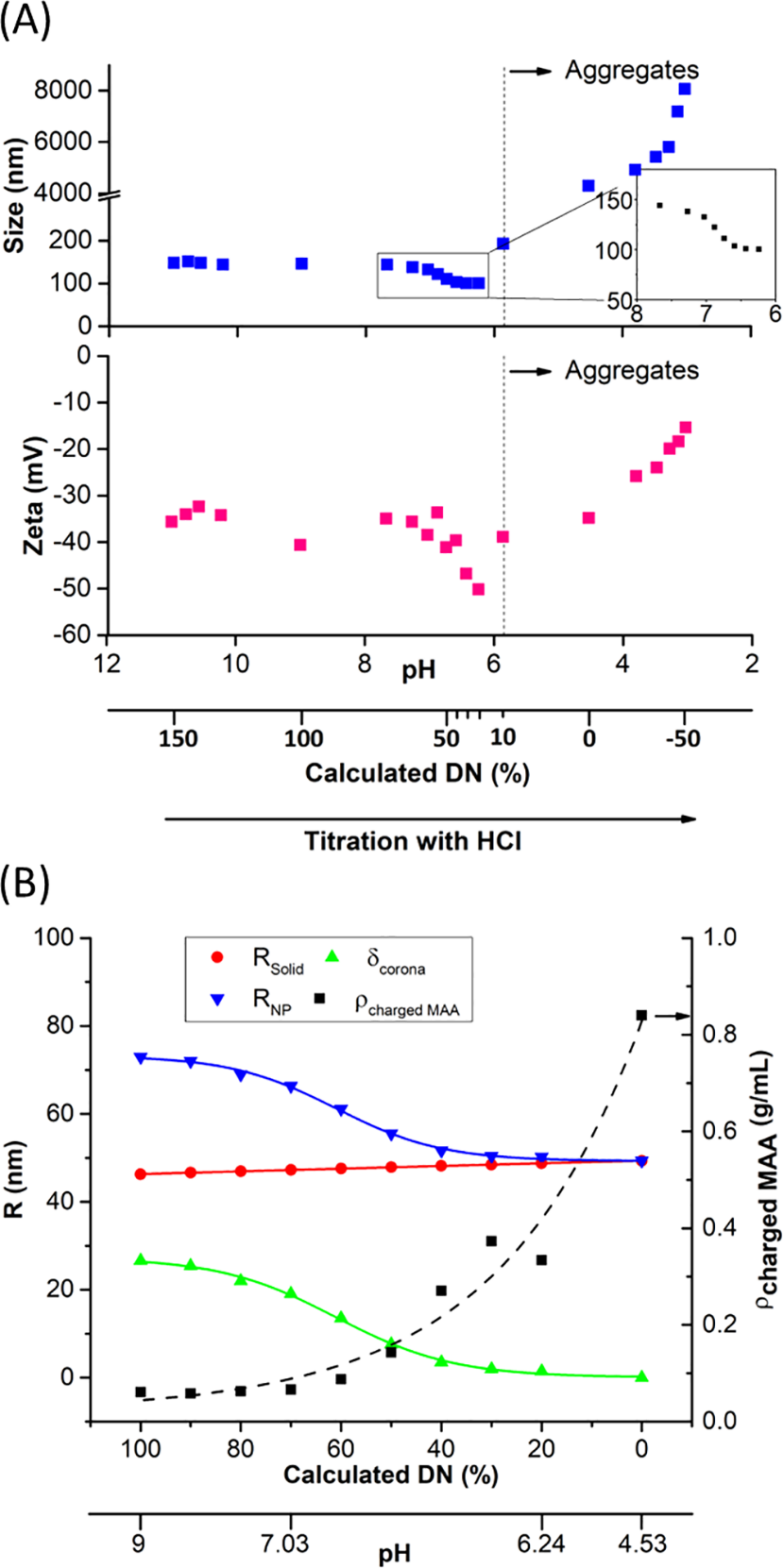
pH responsiveness of S100-stabilized NPs. (A) Change in the NP size and ζ potential in response to HCl titration. (B) Calculated shell density and the change in shell thickness in response to the neutralization of the MAA charge in S100-stabilized NPs.

To examine the pH responsiveness of S100-stabilized NPs, NPs were prepared with excess DN (150%), ionic strength at 125 mM, the core/S100 ratio at 1:1, 10 mg/mL of TMC, and the THF/H_2_O ratio at 25:75 (Table S3). As prepared, the NPs had diameters of 148 nm and a ζ potential of −36 mV (Figure 3A). Increasing NaOH concentrations above 100% DN neither increases the size nor decreases the ζ potential (i.e., makes the NPs less negative). The addition of HCl to the preformed NPs decreases the number of ionized MA units in the hydrated corona. The NP size decreases from 144 to 100 nm over the pH range of 7.7 to 6.2 (see insert in Figure 3A). The p*K*_a_ calculated from this titration curve is 6.7 (Figure S4A), which is similar to the reported p*K*_a_ (6.8) of S100.^34^ Within one-half pH unit below the p*K*_a_, the ζ potential decreases from −35.0 to −50.2 mV. This is not intuitive since at pH values below the p*K*_a_, the MAA units should be less charged, not more charged (i.e., greater negative values of the ζ potential). The increased negative charge in the ζ potential measurement is consistent with the ionized MAA groups in the hydrated corona layer increasing in apparent density as the corona collapses. The ζ potential is a measurement of the apparent charge at the slip plane of the NP surface. That apparent charge is a balance between the actual charge density and the Brinkman permeability of the corona layer.^35^ Below DN = 10%, the NPs have insufficient surface charge to remain colloidally stable; NP size increases due to aggregation (in Figure 3A, note the axis change below DN = 10%, indicating NP aggregation). The opacity of the suspension increases for DN below 10%, also indicating aggregation (Figure S4A).

From the NP size, composition, and response to neutralization, it is possible to calculate the density of polymer chains in the charged corona. We make the following assumptions: (1) the densities of MMA and uncharged MAA are equivalent at 0.84 g/mL, (2) MAA is stoichiometrically protonated with the addition of HCl as shown by the NMR data, (3) the number density of NPs does not change during neutralization, and (4) only ionized MAA contributes to the thickness of corona; also, at 0% DN, the S100 chains are collapsed onto the surface of the NP, and that size is estimated by the sigmoidal asymptote in Figure S4B.

Based on these assumptions, the volume of NPs in the dispersion is given by the volume of an individual NP, *V*_NP,*i*_ times the number of NPs, *n* (eq 3). The subscript *i* denotes a specific DN. The volume of a NP is calculated from its DLS measured size. The total volume is also given by the sum of the volume of all hydrophobic, water-insoluble components, *V*_solid,*i*_, and the volume of the charged corona.
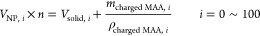
3

The total number of NPs, *n*, is estimated from eq 4. *V*_NP,0_ is the volume of NPs at DN = 0, which is estimated from Figure S4B.
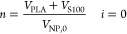
4

*V*_solid,*i*_ includes the volume of PLA, non-ionizable MMA monomers, and uncharged MAAs (eq 5). The volume of each species is calculated using their bulk densities: PLA (1.24 g/mL) and S100 (0.84 g/mL).
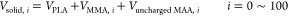
5*m*_charged MAA,*i*_ is the mass of charged MAAs in S100 and can be calculated from eq 6. The molecular weight difference between protonated and deprotonated MAAs is 1.2%; therefore, we neglect the molecular weight change when MAAs are neutralized. *m*_S100_ is the total mass of S100.
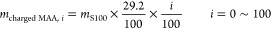
6

This leaves the density of ionized MAA in the corona, ρ_charged MAA,*i*,_ as the only unknown variable. It can be obtained by solving eq 3.

The radius of the hydrophobic solid core R_solid,i_, comprising the hydrophobic core, hydrophobic S100 monomers, and non-ionized S100 monomers, can be calculated from eq 7.
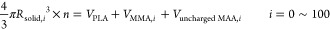
7

The hydrated shell thickness δ_corona,*i*_ can be calculated from eq 8. *R*_NP_ can be measured through DLS.
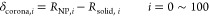
8

The results for the analysis from 0% DN to 100% DN are shown in Figure 3B. The radius of the hydrophobic core is 46 nm as initially precipitated at DN = 100% and increases only by 2 nm as the NP is neutralized to DN = 20%. Experimentally, at DN = 10%, NP aggregation is observed. The thickness contributed by the condensed, non-ionized MA is relatively small. On the other hand, δ_corona,*i*_ decreases by 25 nm during neutralization, which accounts for 89% of the total NP size change. The thickness of the ionized polyelectrolyte layer is a major contributor to the size of the ionized NP. The calculated density of MA in the corona at DN = 100% is 0.061 g/mL, which is consistent with the density of polyelectrolyte polymer brushes reported on silica surfaces (assuming the thickness of corona is 25 nm).^36^ Both *R*_NP_ and δ_corona_ follow similar sigmoidal functions with inflection points at pH 6.9, which is consistent with the calculated p*K*_a_ = 6.7 of S100-NPs. This confirms the responsiveness of the S100 coating to change the size of pre-made NPs.

A dispersion of these S100-stabilized NPs would be exposed to neutral pH at the point of oral administration, a pH of 1–2.5 in the stomach and a pH of 6–7.4 in the intestines.^37,38^ To assess the reversibility of aggregation under acidic conditions, a S100-NP suspension was titrated from pH 9 to pH 3 and then backed up to pH 9 (Figure S4C). The initial size was 146 nm, and after the cycle, the size was 233 nm. The increase in size denotes some degree of irreversible aggregation upon complete neutralization of the MAA corona. However, the redispersion to 232.7 nm is still small enough to obtain the benefit of the higher surface-to-volume effect on enhanced dissolution as described by the Noyes Whitney equation.^39^

### Effect of Different Actives that are Encapsulated

**Figure 4:**
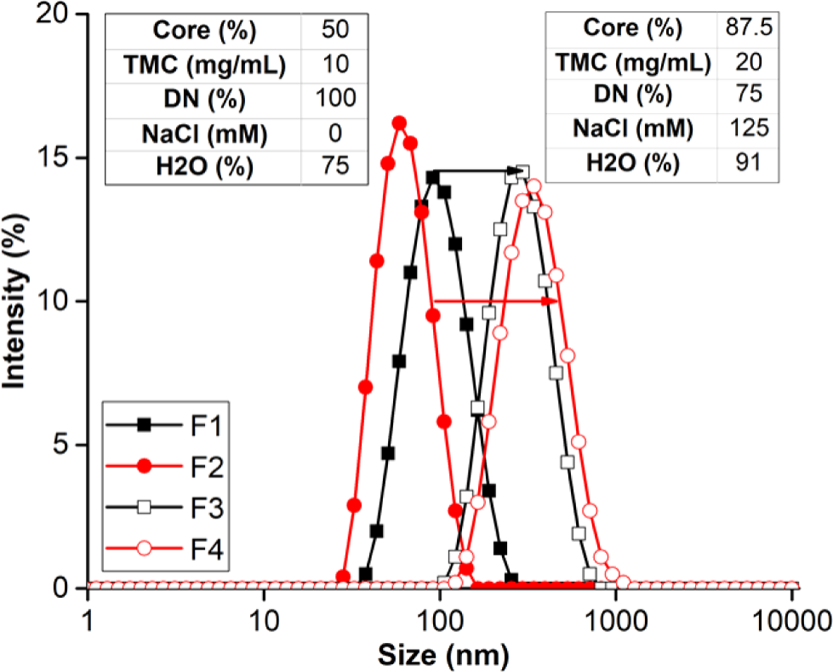
Tuning the size of MAC-coated NPs with an inert core and a small-molecule drug via FNP. Side-by-side comparison of size distribution of S100-coated NPs encapsulating PLA (F1 and F3) and LMN (F2 and F4) given the same formulation variables. Tables on the lefthand and righthand sides of peaks indicate the formulation parameters for F1 and F2 and F3 and F4, respectively.

We have conducted the above study primarily with the model core material PLA. A key question is whether these data and guiding principles will apply to other drug compounds that might be encapsulated by FNP. LMN is a hydrophobic anti-malarial drug and readily encapsulated in NPs via FNP.^17,40^ Here, we demonstrate that both PLA and LMN are formulated into NPs with similar sizes (Figure 4), despite their different molecular weights [PLA (18–28 kDa) versus LMN (528.9 g/mol)]. By varying the percent core, TMC, DN, ionic strength, and supersaturation (% H_2_O), NP sizes below 100 or up to 300 nm can be formed with either core material, as shown in Figure 4. This highlights the translational value of the principles we have presented for NP encapsulation with these polyelectrolyte polymers.

### Observations with Methacrylate-Based Cationic Copolymers and Cellulose-Based Copolymers

While we have focused the detailed discussion above on one type of MAC, that is, S100, the same rules apply to other polyelectrolyte polymers. NP sizes from 91 to 516 nm are formed using the methacrylate-based cationic copolymer (Eudragit RL-PO or RL-PO) by changing formulation parameters (Figure S5A). We have also tested other commercially available pH-responsive MACs (L100-55) and CACs (HP-55, HP-55S, and HPMCAS-HF) as well as S100 to produce NPs encapsulating LMN. The compositions of the polyelectrolyte polymers are given in Table 1. Surprisingly, despite different backbones, molecular weights, or wt % of acid groups, similar sizes (100–200 nm) of NPs were formulated at the core/copolymer mass ratio of 87.5:12.5, 100% DN, 10 mg/mL of TMC, and 75% H_2_O (Figure S5B). This provides a good starting point for further tuning to the size of interest using commonly used pH-responsive polymers.

Release profiles of LMN from the above NPs were dependent on the dissolution pH of the stabilizing polyelectrolytes (Figure S5C). Due to its hydrophobic nature, the EE of LMN was near 100%, regardless of the formulations. The pH values reported by the manufacturers for the dissolution of enteric polymer films are pH = 5 for HP-55, pH = 5.5 for L100-55, HP-55s, and pH = 7 for S100. NPs stabilized with S100 release 83% of the payload, while NPs stabilized with HP-55 achieve almost complete release (97%) of LMN within 6 h in an intestinal buffer made with 10% v/v FaSSGF and 90% v/v FaSSIF.

To produce large-scale oral nanomedicines, formulations must be stable throughout the downstream processing: dialysis, tangential flow filtration (TFF), freeze-drying, and resuspension. We have prepared an 80 mL batch size of S100-stabilized NP formulation encapsulating BDP 650/665 (1.2 wt % of core) for an animal study (unpublished). These particles were stable throughout the downstream processing up to the resuspension of lyophilized powder (Figure S5D). Although this demonstrates the stability of S100-stabilized NPs, one will have to individually optimize downstream processing depending on the formulation and application.

The valency of the salt is also important in the formulation. As we have shown, increasing concentrations of NaCl produce larger NPs. Using divalent salts also decreases the volume of the hydrophilic domain, but at the cost of introducing attractive inter-chain and inter-particle associations and aggregation (Figure S5E). This is similar to the effect of divalent ions on the precipitation of anionic polymers.^41,42^

## Conclusions

In summary, we have showcased the capability to control the size of particles using a widely used MAC (Eudragit S100) by changing the (1) DN; (2) ionic strength; (3) core-to-stabilizer ratio; and (4) TMC in the precipitation. The rules for controlling the sizes of polyelectrolyte-stabilized NPs also applied to the formation of NPs stabilized by other Eudragits, including cationic Eudragits, and CACs. The S100-stabilized NPs show pH-responsive changes in size, which were shown to be associated with changes in the thickness of the polyelectrolyte corona. While a model PLA core material was used for most of the studies, the size control of NPs encapsulating the small-molecule core LMN showed that the rules apply to a broad spectrum of hydrophobic active pharmaceutical ingredients (APIs) that one might want to encapsulate. The release of API from the NP core was largely dependent on the pH responsiveness of polymers. We expect researchers in the pharmaceutical field to take advantage of these results to tune the size of particles and to scale up the formulations that might be used for the development of future drug delivery systems.

## Supporting Information Available

DLS data of S100; original ^13^C NMR spectra; pH and size change of S100-stabilized NPs as a function of DN; size change of RL-PO-stabilized NPs as a function of ionic strength and TMC; size of LMN-encapsulated NPs stabilized with different types of polyelectrolytes; the release of LMN from NPs stabilized with different types of polyelectrolytes; and the size change of S100-stabilized NPs as a function of ionic strengths and different valencies of salts (PDF)
