# Supplementary material for: Formulation of pH-Responsive Methacrylate-Based Polyelectrolyte-Stabilized Nanoparticles for Applications in Drug Delivery
Source: ACS Appl Nano Mater. 2022 Nov 24;5(12):18770–8. doi: 10.1021/acsanm.2c04539 (PMC9791616; doi:10.1021/acsanm.2c04539)
Supplement: Supplementary file 1 — an2c04539_si_001.pdf [file an2c04539_si_001.pdf]

Formulation of pH-responsive Methacrylate-based Polyelectrolyte Stabilized Nanoparticles for Applications in Drug Delivery

Bumjun Kim[a], Dawei Zhang[a], Madeleine S. Armstrong[a], István Pelczer[b], and Robert K. Prud'homme[a]*

[a]Department of Chemical and Biological Engineering, Princeton University, Princeton, New Jersey 08544, United States

[b]Department of Chemistry, Princeton University, Princeton, New Jersey, 08544, United States

*prudhomm@princeton.edu

Supporting Information

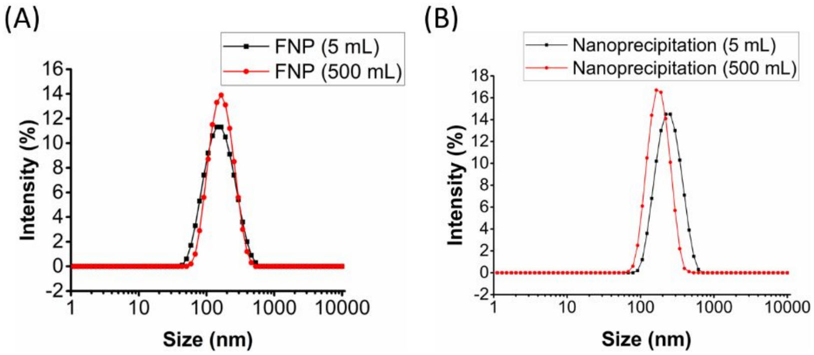

Figure S1. Size distributions of NPs produced at 5 mL and 500 mL scales via FNP (A) or conventional nanoprecipitation (B). PLA and Eudragit S100 were dissolved in THF at 5mg/mL, respectively, then this organic stream was mixed with aqueous solution stream 125 mM NaCl and 1 equivalent amount of NaOH relative to carboxyl group in S100 via FNP or conventional nanoprecipitation process.

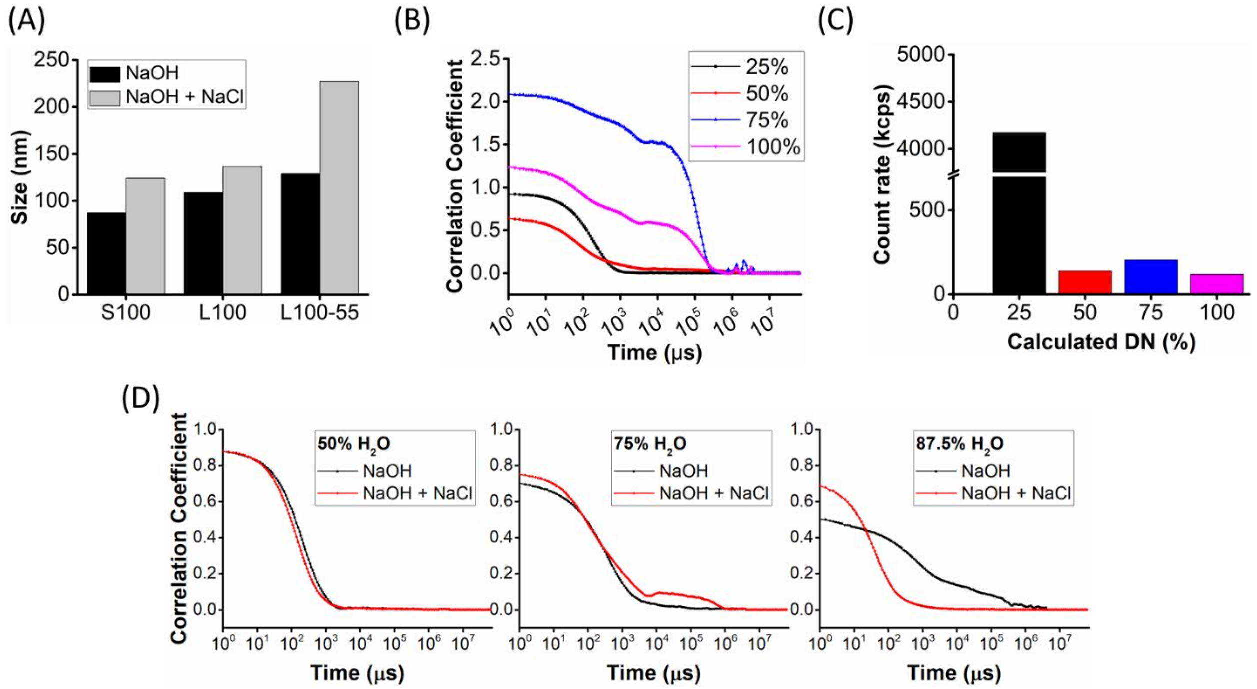

Figure S2. (A) Size of NPs stabilized with different MACs with or without salt screening. PLA was used as a hydrophobic core and mass ratios of PLA to MACs were 1:1. (B) Correlogram and (C) count rate of S100 (5mg/mL) precipitated via FNP at different degrees of neutralization in the solvent mixture of 25% (v/v) THF and 75% (v/v) water. (D) Correlogram of S100 (2.5mg/mL) precipitated at different levels of supersaturation via FNP. For all the conditions, degree of neutralization was 100%. Total salt concentration in anti-solvent stream was maintained at 125 mM for (A), (B), (C) and (D).

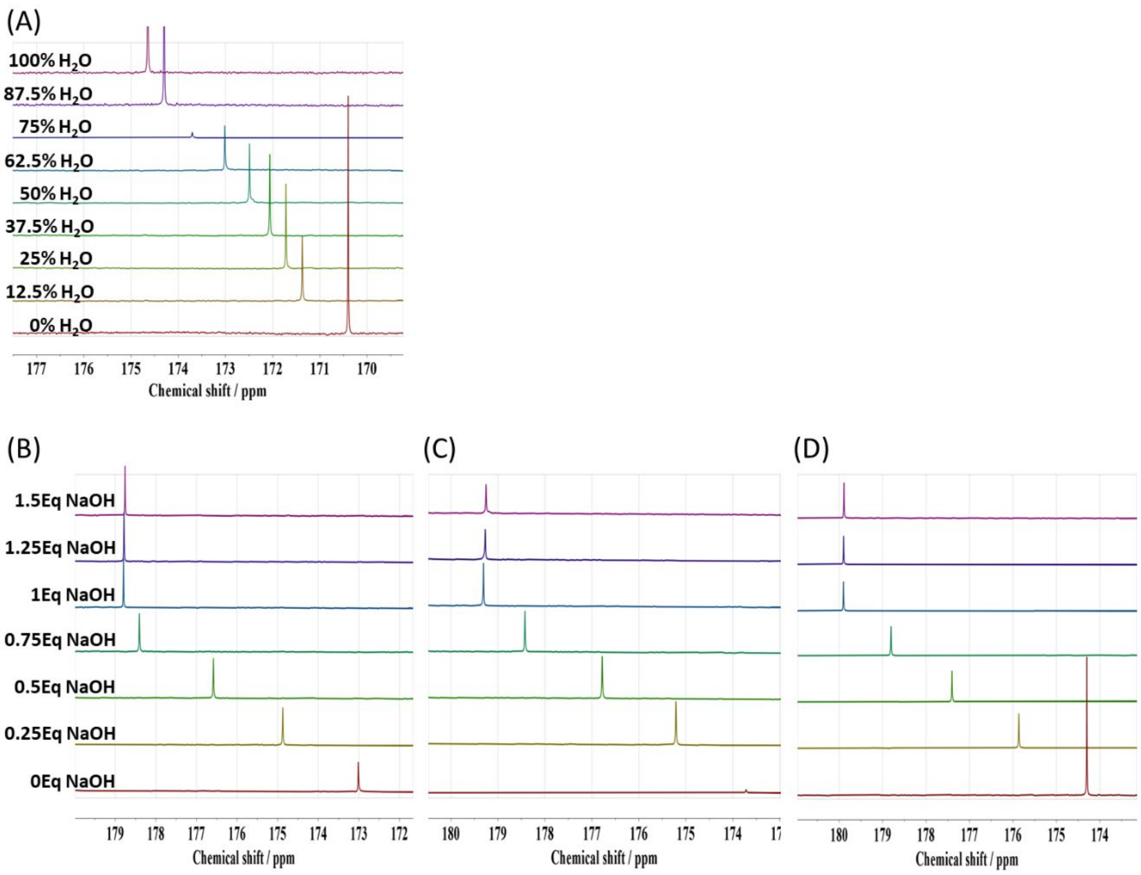

H_2O % v/v	**C**OOH, chemical shift / ppm
100%	174.65
87.5%	174.3
75%	173.7
62.5%	173.01
50%	172.49
37.5%	172.06
25%	171.72
12.5%	171.37
0%	170.4

	**C**OOH, chemical shift / ppm		
**NaOH / eq.**	**62.5% v/v H_2O**	**75% v/v H_2O**	**91% v/v H_2O**
1.5	178.76	179.25	179.89
1.25	178.78	179.27	179.90
1	178.80	179.31	179.90
0.75	178.41	178.42	178.81
0.5	176.58	176.78	177.40
0.25	174.87	175.21	175.86
0	173.01	173.72	174.30

Figure S3. (A) ^{13}C NMR spectra of COOH of MAAs as a function of percent water. (B) ^{13}C NMR spectra of COOH of MAAs as a function of degree of neutralization in a solvent mixture of water to THF ratio at (B) 62.5:37.5, (C)75:25, and (D) 91:9, respectively

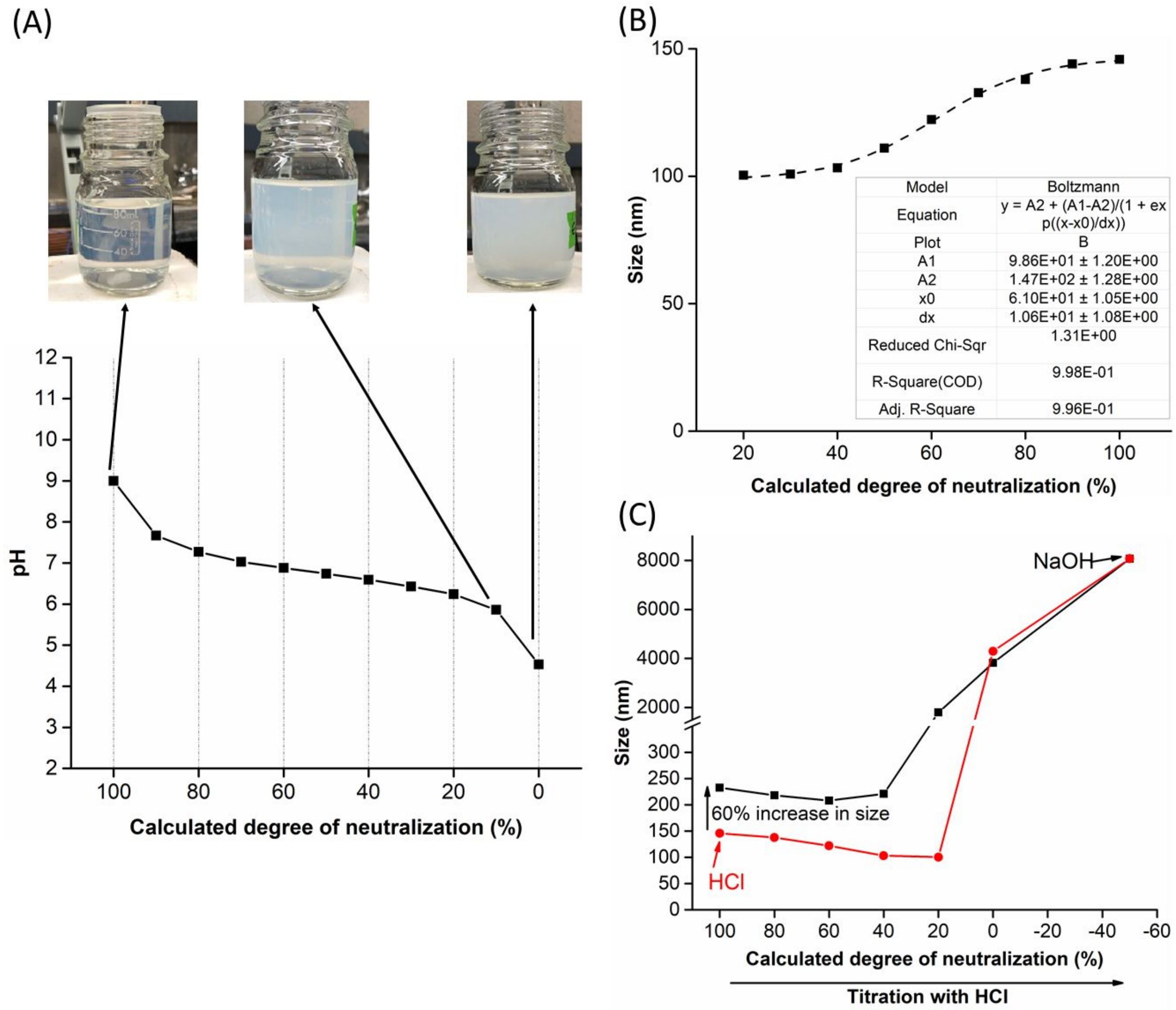

Figure S4. (A) Titration of S100-stabilized NPs with HCl. (B) Fitting the size of S100-stabilized NPs produced at different degree of neutralization to a sigmoidal curve. (C) Titrating S100-stabilized NPs with HCl until 50% excess HCl is added (-50% on x-axis) compared to the number of MAAs in S100. Then, NaOH was added back to 100% DN to fully ionize S100-stabilized NPs.

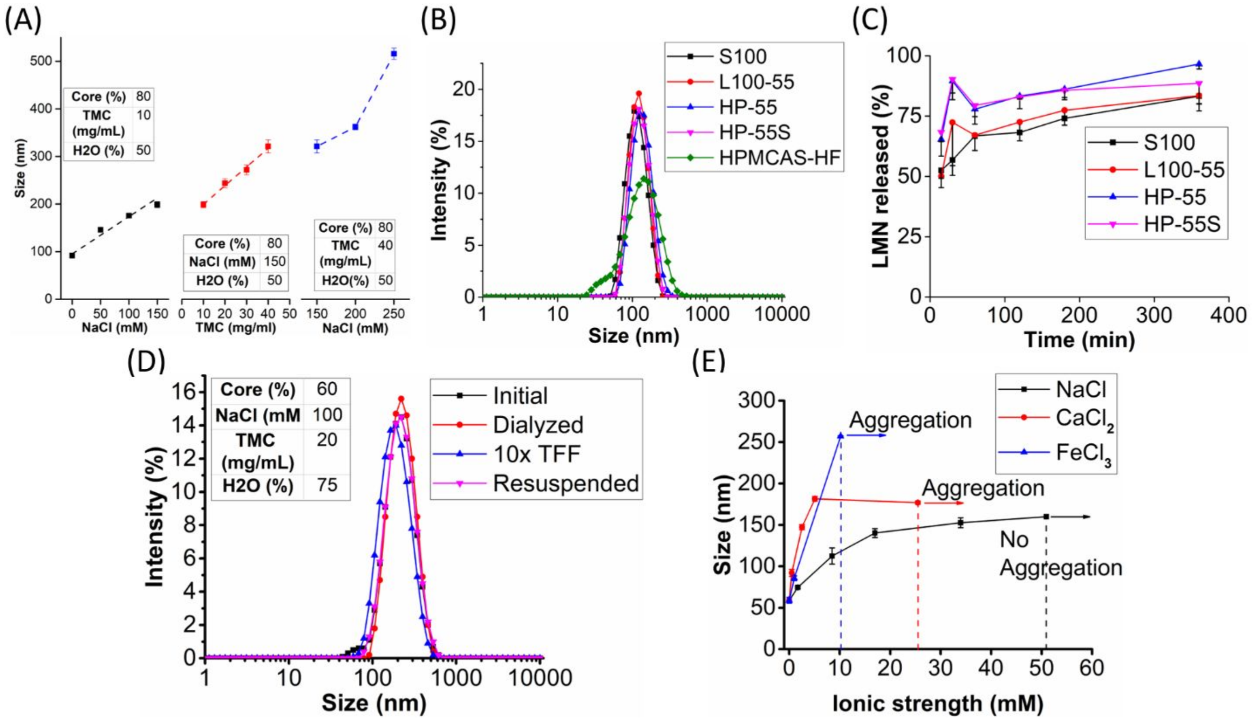

Figure S5. (A) Controlling the size of RL-PO coated NPs via FNP. PLA and RL-PO were dissolved in THF at 4:1 mass ratio and loaded on solvent stream, which was impinged against 1 part of water. Salt concentration and TMC were changed as indicated. (B) Size distribution of LUM encapsulating NPs coated with different enteric coating. The mass ratio of LUM to enteric polymers was 7:1. TMC was 10 mg/mL. The NaOH concentrations were stoichiometrically equivalent to concentrations of ionizable groups in each polymer. No extra NaCl was added. (C) The release of LUM was measured in a mixture of FaSSGF and FaSSIF. NPs were first incubated in FaSSGF for 30 mins, then, 10-fold diluted in FaSSIF. Samples were collected at indicated time points. (D) Change of size distributions of BDP 650/665 encapsulated NPs throughout the downstream processing. Only 1.2 wt% of core is BDP 650/665 and the rest is PLA. (E) Changing the size of particles as a function of ionic strengths of different valency of salts.

Table S1. Chemical structures of small molecules and polymers used in this paper.

Name	Chemical Structure
Lumefantrine	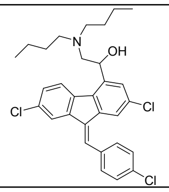
PLA	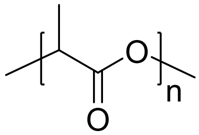
Eudragit S100	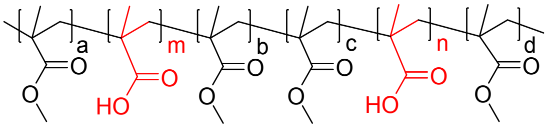<br>(a+b+c) : (m+n) = 2 : 1
Eudragit L100	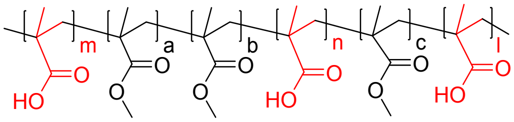<br>(a+b+c) : (m+n+l) = 1 : 1
Eudragit L100-55	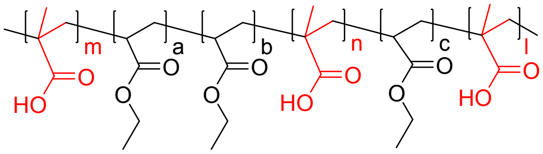<br>(a+b+c) : (m+n+l) = 1 : 1
HP-55<br>HP-55s	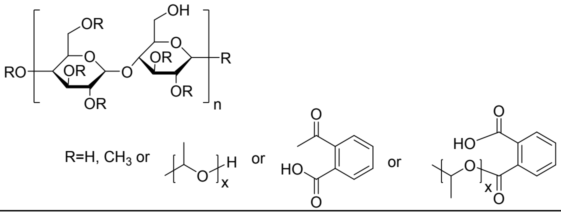
HPMCAS-HF	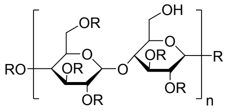<br>R=H, CH_3 or —$CH_2CH(CH_3)OH$<br>—$COCH_3$ —$COCH_2CH_2COOH$<br>—$CH_2CH(CH_3)OCOCH_3$<br>—$CH_2CH(CH_3)OCOCH_2CH_2COOH$
Eudragit RL-PO	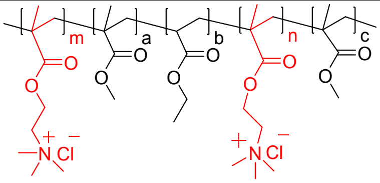<br>b:(a+c):(m+n) = 1:2:0.2

Table S2. Physicochemical properties of NPs in **Figure 1**.

Figure	Calculated DN (%)	NaCl (mM)	Core (%)	TMC (mg/mL)	%v/v H_2O	Size ± SEM (nm)	PDI ± S.E.M	Zeta ± SD (mV)
**A**	25	125	50	10	75	180 ± 11	0.18 ± 0.01	-34 ± 7
	50					191 ± 2	0.23 ± 0.01	-34 ± 6
	75					159 ± 5	0.24 ± 0.01	-31 ± 10
	100					136 ± 7	0.21 ± 0.01	-38 ± 8
**B**	100	5	50	10	75	75 ± 1	0.23 ± 0.03	-34 ± 3
		12				84 ± 1	0.23 ± 0.02	-35 ± 2
		24				101 ± 10	0.25 ± 0.04	-35 ± 3
		47				110 ± 5	0.25 ± 0.01	-38 ± 3
		94				121 ± 6	0.21 ± 0.01	-40 ± 3
		141				132 ± 5	0.20 ± 0.01	-34 ± 3
		189				138 ± 6	0.20 ± 0.01	-32 ± 4
**B***	100	1	50	10	91	75 ± 1	0.26 ± 0.01	-25 ± 4
		2				82 ± 1	0.24 ± 0.01	-21 ± 4
		8				113 ± 1	0.20 ± 0.01	-31 ± 3
		17				140 ± 3	0.20 ± 0.01	-39 ± 4
		34				153 ± 4	0.21 ± 0.02	-26 ± 6
		51				160 ± 4	0.20 ± 0.01	-32± 6
		85				172 ± 1	0.18 ± 0.02	-27 ± 3
		170				182 ± 4	0.17 ± 0.01	-30 ± 2
**C**	100	125	25	10	75	111 ± 4	0.28 ± 0.01	-26 ± 6
			37.5			129 ± 5	0.25 ± 0.03	-32 ± 8
			50			134 ± 5	0.19 ± 0.01	-35 ± 2
			62.5			143 ± 6	0.19 ± 0.01	-37 ± 2
			75			151 ± 2	0.16 ± 0.01	-35 ± 1
			87.5			169 ± 5	0.16 ± 0.02	-38 ± 2
**D**	100	125	50	10	75	142 ± 3	0.20 ± 0.01	-38 ± 8
				20		191 ± 4	0.18 ± 0.01	-38 ± 1
				30		234 ± 3	0.15 ± 0.01	-40 ± 0
				40		262 ± 2	0.14 ± 0.01	-40 ± 0
**E**	75	125	75	10	91	185 ± 1	0.17 ± 0.01	-35 ± 8
				20		270 ± 1	0.15 ± 0.01	-40 ± 1
				30		393 ± 5	0.16 ± 0.00	-43 ± 0
				40		454 ± 6	0.11 ± 0.01	-44 ± 1

*This experiment was not replicated. Error bar is expressed as ± S.D.

Table S3. Physiochemical properties of NPs in **Figure 3A and Figure S4**.

Calculated DN (%)	NaCl (mM)	Core (%)	TMC (mg/mL)	%v/v H_2O	pH	Size (nm)	PDI	Zeta ± SD (mV)
**150**					11.0	148	0.19	-36 ± 7
**140**					10.9	N/A	N/A	N/A
**130**					10.8	152	0.18	-34 ± 10
**120**					10.6	148	0.16	-32 ± 8
**110**					10.2	144	0.17	-34 ± 8
**100**					9.0	146	0.17	-41 ± 8
**90**					7.7	144	0.18	-35 ± 8
**80**					7.3	138	0.17	-36 ± 8
**70**					7.0	133	0.15	-38 ± 8
**60**					6.9	122	0.16	-34 ± 8
**50**					6.7	111	0.16	-41 ± 8
**40**					6.6	103	0.15	-40 ± 8
**30**					6.4	101	0.13	-47 ± 8
**20**	125	50	10	75	6.2	100	0.12	-50 ± 8
**10**					5.9	193	0.48	-39 ± 8
**0**					4.5	4294	0.63	-35 ± 8
**-10**					3.8	4920	0.86	-26 ± 8
**-20**					3.5	5422	1.00	-24 ± 8
**-30**					3.3	5807	1.00	-20 ± 8
**-40**					3.1	7194	1.00	-18 ± 8
**-50**					3.0	8075	1.00	-15 ± 8
**0**					5.5	3821	1.00	-38 ± 4
**20**					6.7	1795	1.00	-31 ± 2
**40**					7.0	221	0.15	-36 ± 3
**60**					7.1	208	0.11	-38 ± 5
**80**					8.9	218	0.09	-37 ± 1
**100**					10.7	233	0.09	-39 ± 3
